# Similarity-assisted variational autoencoder for nonlinear dimension reduction with application to single-cell RNA sequencing data

**DOI:** 10.1186/s12859-023-05552-1

**Published:** 2023-11-14

**Authors:** Gwangwoo Kim, Hyonho Chun

**Affiliations:** 1 Graduate School of Data Science, Korea Advanced Institute of Science and Technology (KAIST), Daejeon, Republic of Korea; 2 Department of Mathematical Sciences, Korea Advanced Institute of Science and Technology (KAIST), Daejeon, Republic of Korea

**Keywords:** Deep learning-based nonlinear dimension reduction, Variational autoencoder, Uniform manifold approximation and projection, Single-cell RNA sequencing data, Covariate effect

## Abstract

**Background:**

Deep generative models naturally become nonlinear dimension reduction tools to visualize large-scale datasets such as single-cell RNA sequencing datasets for revealing latent grouping patterns or identifying outliers. The variational autoencoder (VAE) is a popular deep generative method equipped with encoder/decoder structures. The encoder and decoder are useful when a new sample is mapped to the latent space and a data point is generated from a point in a latent space. However, the VAE tends not to show grouping pattern clearly without additional annotation information. On the other hand, similarity-based dimension reduction methods such as t-SNE or UMAP present clear grouping patterns even though these methods do not have encoder/decoder structures.

**Results:**

To bridge this gap, we propose a new approach that adopts similarity information in the VAE framework. In addition, for biological applications, we extend our approach to a conditional VAE to account for covariate effects in the dimension reduction step. In the simulation study and real single-cell RNA sequencing data analyses, our method shows great performance compared to existing state-of-the-art methods by producing clear grouping structures using an inferred encoder and decoder. Our method also successfully adjusts for covariate effects, resulting in more useful dimension reduction.

**Conclusions:**

Our method is able to produce clearer grouping patterns than those of other regularized VAE methods by utilizing similarity information encoded in the data via the highly celebrated UMAP loss function.

## Background

Deep generative models [1–3] have been naturally utilized for large-scale biological datasets such as RNA-sequencing data [4–8]. The generative models assume lower-dimensional latent random variables and map them to high-dimensional data. Under these models, the inverse mapping of high-dimensional data to lower-dimensional embeddings can be used as a nonlinear dimension reduction tool.

Among the deep generative models, the variational autoencoder (VAE) [9] and the generative adversarial network (GAN) [10] have been widely investigated. In a VAE, probabilistic hierarchical models are used with the encoder/decoder structure. The encoder is an approximation of the posterior distribution, which maps high-dimensional data to the lower-dimensional embedding, and the decoder is the data distribution function that matches the lower-dimensional embedding to the high-dimensional data. In a GAN, generative mapping is inferred by optimizing a min-max problem. GAN in its original form is not equipped with an encoder because its best use is in generating realistic data. Although it can be trained with an auxiliary encoder, as in [11, 12], we do not pursue GAN in this work because our study showed that, compared to GAN, VAE tends to yield a more stable latent representation.

On the other hand, similarity-based dimension reduction tools show excellent performance and have been widely adopted, as evidenced by t-distributed stochastic neighbor embedding (t-SNE) [13] and uniform manifold approximation and projection (UMAP) [14]. These methods present a clear grouping structure, although it is not equipped with the encoder/decoder structure, which makes new sample extensions of the methods challenging.

To bridge this gap, we propose a new method, a similarity-assisted variational autoencoder (saVAE), which adopts similarity information in the framework of the VAE. We pursue this by adding pull-push regularization to the evident lower bound of the likelihood function. Our method produces a powerful latent representation superior to both VAE and similarity-based approaches. Besides this, for single-cell RNA sequencing data applications, a meaningful group pattern can be easily disguised by many other covariates such as donor or batch effects. We thus extend our approach to the conditional VAE (CVAE) to adjust for such covariate effects.

Our contributions are (1) formulating an objective function that combines similarity-based and model-based approaches to promote a type of dimension reduction that best reflects hidden structures in data without informative priors, (2) adjusting for covariates in the dimension reduction step so that the meaningful lower dimensional representation is not disguised by the covariate effects, and (3) comparing our methods to state-of-art dimension reduction methods.

## Results

### Overview of saVAE

**Fig. 1:**
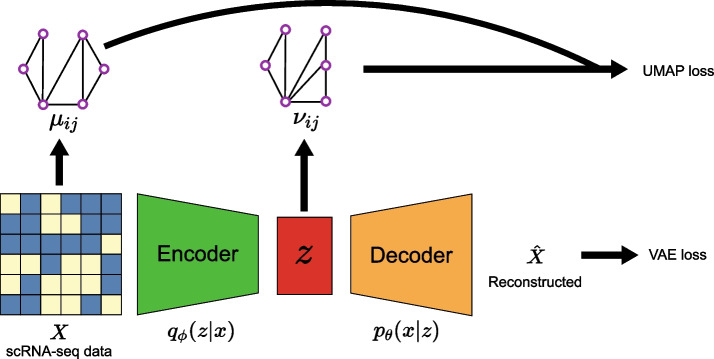
Overview of saVAE. Our saVAE connects the VAE and UMAP objective functions by utilizing the expected UMAP loss function, which yields a dimension reduction result with a more informative grouping structure

Our saVAE aims at combining the objective functions of VAE and UMAP (see Methods). The VAE training requires estimating two sets of parameters involved with the encoder and decoder. On the other hand, UMAP is a non-parametric method to directly infer individual embeddings. Thus it is not trivial to integrate UMAP into the VAE framework. To resolve this problem, we connect the two objective functions by utilizing the expected UMAP loss function, because the expectation of the UMAP loss is the function of the decoder parameters. Our model is summarized in Fig. 1. There are several important computational challenges in combining these two methods, such as implementing batching schemes and balancing the convergence of the two approaches. These issues are further discussed in the Methods section.

When there are external covariates available, they need to be included in the model to remove extra sources of variation. For example, in a single-cell RNA sequencing data analysis, differences in batches or donors may induce extra groupings. To address the covariates, we utilize CVAE, which models covariate effects with an additional multi-layered perceptron (MLP). We then regularize the CVAE with the expected UMAP loss function, called saCVAE.

In summary, our method has the following advantages: (1) saVAE bridges the parametric method with the nonparametric one by computing the posterior expectation of the nonparametric latent variables. (2) By using batches with carefully sampled pairs, saVAE becomes scalable to handle large-scale datasets. (3) Differences in algorithmic convergence are balanced using a regularization parameter and by adopting proportional updates, which removes extra groups in inferred latent structures.

### Synthetic data analysis

**Fig. 2:**
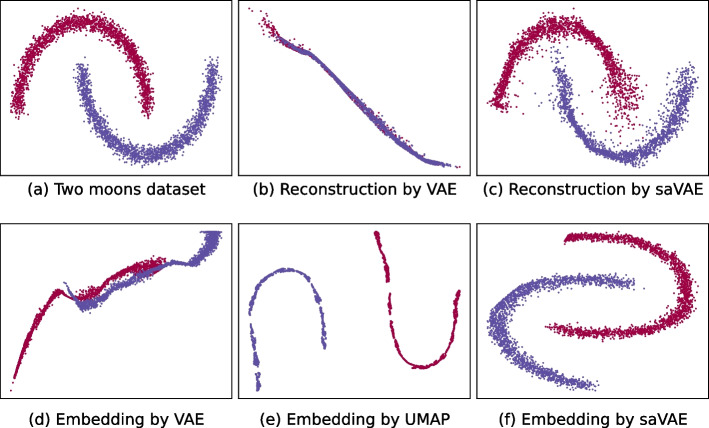
Method comparison using two moons dataset. The saVAE performs better than either VAE or UMAP in terms of finding meaningful latent embeddings and producing accurate reconstruction

We first present the synergies obtained by combining VAE and UMAP with a simulated dataset. The dataset is generated following the sklearn API [15], called the two moons dataset. As shown in Fig. 2a, the dataset consists of two interweaving half circles in a two-dimensional space. Each half-circle has 2000 points and becomes a class. The sklearn method sklearn.datasets.make_moons generates the exact same dataset, with $\text{noise} = 0.05$ and $\text{random}\_\text{state} = 42$.

The embeddings inferred from VAE, UMAP, and saVAE are presented in Fig. 2d–f, respectively. The VAE learns neither meaningful latent representation nor accurate reconstruction. On the other hand, the UMAP yields a latent representation revealing two half-circles but does not show the interweaving features between them. Our saVAE captures the interweaving half-circles accurately. Unlike UMAP, VAE and saVAE can produce the reconstructed data points via the inferred decoder. The reconstructed points are compared in Fig. 2b, c. The saVAE’s reconstruction is much closer to the original than VAE’s. This simulation study clearly shows the benefits of saVAE, which are that it inherits the structure of VAE while incorporating the power of the UMAP method.

### MNIST data analysis

The deep clustering via a Gaussian-mixture variational autoencoder with graph embedding (DGG) [16] is perhaps the most similar approach to our saVAE: it inferred the latent representation using the variational deep embedding (VaDE) [17] model with distance regularization. A detailed discussion of the approach is in the Related Works section. We compare the embeddings from DGG and saVAE as well as UMAP to illustrate the differences among the methods.

The MNIST dataset consists of 60k digit images from 0 to 9. Each data point is in the form of a 28 $\times$ 28 matrix whose entries are values in the set [0,255]. Dividing the whole dataset by the maximum value 255, all values are in [0,1]. We binarize the resulting values to use a Bernoulli model (6).

**Fig. 3:**
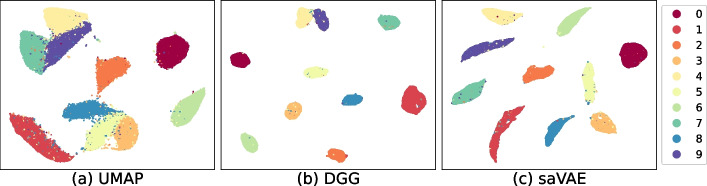
Embeddings from MNIST dataset. **a** The UMAP in general separated the groups accurately except for two clusters that needed to be further separated. **b** The DGG formed almost perfect groups in the latent space, yet the gap between the two clusters (digits 4 and 9) was still slim. **c** The saVAE showed perfect groups in the latent space including digits 4 and 9

In Fig. 3, the UMAP in general separates the groups accurately (Fig. 3a), except for two clusters that needed to be further separated. The DGG forms almost perfect groups in the latent space (Fig. 3b), yet the gap between the two clusters (digits 4 and 9) is still slim. The saVAE shows perfect groups in the latent space, including digits 4 and 9 (Fig. 3c).

### Single-cell RNA sequencing data analysis when there is no covariate

We compare our saVAE to UMAP, scvis [18], single-cell Variational Inference (scVI) [6, 19], and Ivis [20] as these approaches have available code with excellent performance. The following two datasets are used: (1) cortex [21] and (2) peripheral blood mononuclear cells (PBMC) [22]. The cortex dataset is from the cortex cells of mice. The number of cells is 3005 with seven cell types. The PBMC dataset is one of the most commonly used biological datasets. For example, the dataset is used for immunology [23], regenerative medicine [24], and drug discovery [25]. The number of cells is about 12k, and there are nine cell types. We remark that the PBMC dataset is comprised of two batches of PBMCs (4K PBMCs and 8K PBMCs), but we did not use the batch information because its effect was negligible (see Additional file 1: Figure S1). For both datasets, we used 1200 genes selected by using the highest varying genes (HVGs) method [26]. We then compared the inferred embeddings visually as well as quantitatively.

For quantitative evaluation, we compare clustering accuracy after performing clustering on inferred embeddings. The embedding with higher clustering accuracy is accepted as a better one. As clustering methods, we consider Kmeans [27] and DBSCAN [28] methods. The Kmeans method is a widely used clustering algorithm, but it can capture only spherically shaped clusters. To avoid this limitation, DBSCAN is utilized because it is another well-known clustering method capable of capturing non-spherical clusters. We applied both clustering algorithms to inferred latent embeddings and reported the better clustering result. Here, clustering performance is compared using the average of the Adjusted Rand Index (ARI) [29] and Normalized Mutual Information (NMI) [30]. In this way, the clustering comparison does not depend on the choice of clustering method. The DBSCAN heavily depends on its parameters (eps and min sample). Thus, we searched for parameters that yield the highest average of ARI and NMI.

**Fig. 4:**
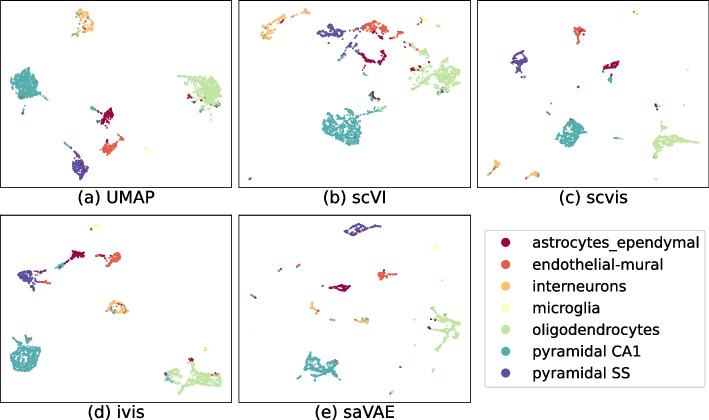
Embeddings from cortex dataset. Compared to other methods, saVAE and scvis show better performance in separating groups

**Fig. 5:**
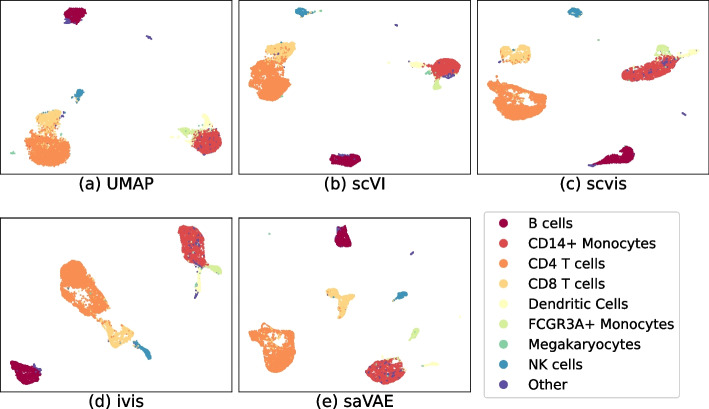
Embeddings from PBMC dataset. The embeddings from our saVAE are superior to those from the other two methods in terms of establishing clusters: CD8 T cells are separated from CD4 T cells, and Dendritic cells and FCGR3A+ Monocytes move away from CD14+ Monocytes

**Fig. 6:**
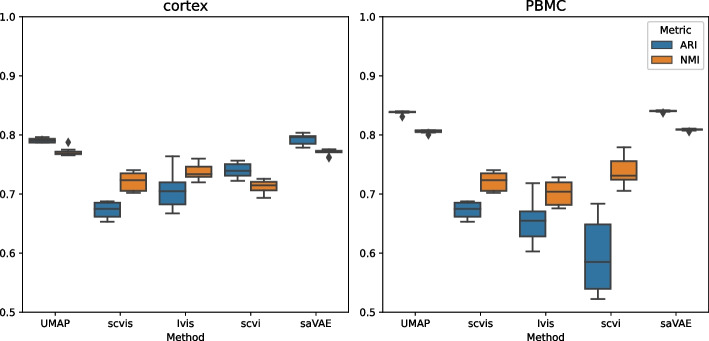
Performance comparison. Our saVAE performs better than UMAP, scvis, Ivis, and scVI. The box plots were created from 10 trials

The embeddings using the cortex and the PBMC datasets are presented in Figs. 4 and 5, respectively. In the cortex data, saVAE shows a clear separation of groups. In the PBMC dataset, the embeddings from our saVAE are superior to those from the other two methods in terms of establishing clusters: CD8 T cells are separated from CD4 T cells, and Dendritic cells and FCGR3A+ Monocytes move away from CD14+ Monocytes. The findings from visual inspection are confirmed in Fig. 6, where our saVAE showed higher ARI and NMI values than UMAP, scvis, Ivis, and scVI.

### Single-cell RNA sequencing data analysis when there are covariates

We compare our method to others using single-cell RNA sequencing datasets when covariates are available. The following two datasets are used: (1) the retina [31] and (2) the heart cell atlas [32] datasets. The retina dataset consists of bipolar cells that exist between photoreceptors and ganglion cells. It contains 20k cells with fifteen cell types in two batches. The original heart cell atlas data consists of 486k cells with fourteen donors of age 40–75. We randomly sample 20k cells and use 4 covariates (cell source, donor, percent mito, and percent ribo). The first two covariates are categorical variables with 4 and 14 levels, respectively, and the others are continuous ones.

**Fig. 7:**
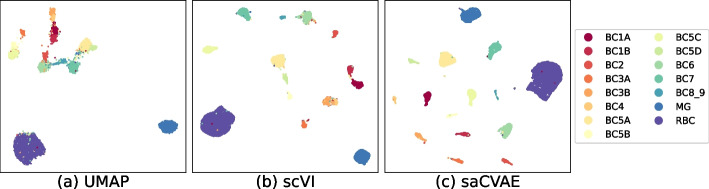
Embeddings from retina dataset The UMAP fails to separate the cell groups. The scVI shows better separation, but there remain several groups that need to be pushed away. The saCVAE completely separates cell types, e.g., BC3A and BC3B

**Fig. 8:**
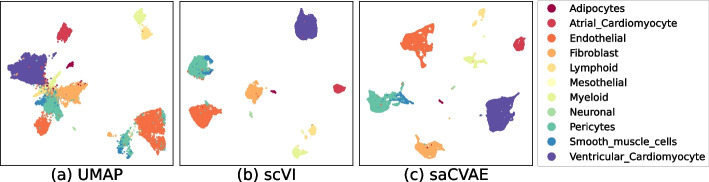
Embeddings from heart cell atlas dataset. The UMAP fails to separate the cell groups. The scVI groups a part of neuronal cells as endothelial cells, whereas saCVAE correctly separates them

**Fig. 9:**
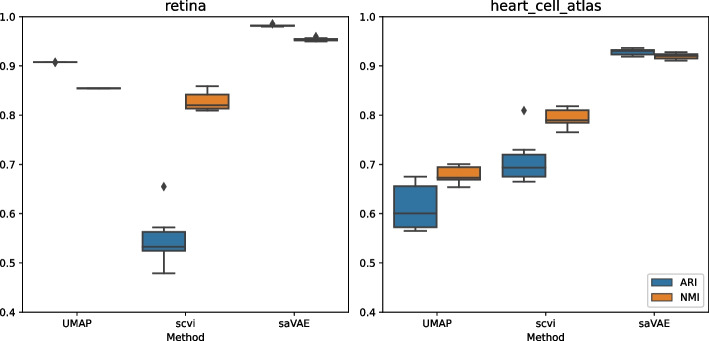
Performance comparison. Our saCVAE formulation effectively eliminates the covariate effects, resulting in a better cell-type clustering performance. The box plots were created from 10 trials

When fitting saCVAE, the CVAE framework is first applied to the training data. The resulting latent embeddings are then used for computing the similarity weights of the UMAP loss function. The details of saCVAE are in the Method section. Among other existing methods, scVI is the only one that adjusts for covariate effects. We thus compare our approach to scVI, not to scvis or Ivis.

Figures 7 and 8 show that embeddings from UMAP, scVI, and saCVAE, where saCVAE tends to group cell types more accurately. In the retina dataset, saCVAE completely separates BC3A and BC3B cells. In the heart cell atlas dataset, scVI groups a part of neuronal cells as endothelial cells, whereas saCVAE correctly separates them. Figure 9 demonstrates this finding with the accuracy measures. Our saCVAE yields much higher ARI and NMI than the other methods.

## Conclusion

In this work, we proposed saVAE, a variant of VAE reflecting the similarity of data in the latent space. Moreover, we extended our approach to CVAE to adjust for biological covariate effects. Finally, we illustrated that saVAE is superior to other nonlinear dimension reduction methods through the various applications to synthetic and real-world datasets. However, saVAE heavily relies on the quality of a similarity table, so the distance metric needs to be selected carefully. Furthermore, our saVAE has higher computation complexity ($O(N^2)$) than a vanilla VAE (*O*(*N*)) as it unifies two different approaches. Here *N* represents the number of samples. To alleviate this problem, we used computational techniques of the negative sampling and the update ratio balancing. In the case of saCVAE, it is a two-stage method that may severely depend on the performance of the first-stage approach. When the first stage incurs bad errors, the second stage cannot correct that. Our future work will deal with the mentioned challenges.

## Methods

### VAE

VAE is an approach for approximating a generative model. A typical generative model uses a latent variable *z* and an observable variable *x*, and the probability density function of $p_{\theta }(x)$ is obtained by $\int p_{\theta }(x|z) p_{\theta }(z) dz$. Often, the marginal probability distribution involves intractable integration; the variational lower bound is also utilized.

1 $$\begin{aligned} \log p_{\theta }(x) \ge \mathbb{E}_{q_{\phi }(z|x)}\log \frac{p_{\theta }(z,x)}{q_{\phi }(z|x)} = \mathbb{E}_{q_{\phi }(z|x)} \log p_{\theta }(x|z) - \mathbb{E}_{q_{\phi }(z|x)} \log \frac{q_{\phi }(z|x)}{p_{\theta }(z)} \end{aligned}$$

Here $q_{\phi }(z|x)$ is a variational distribution that approximates the posterior distribution $p_{\theta }(z|x)$. This lower bound is called the evidence lower bound (ELBO).

The Monte Carlo approximation [33] is widely adopted to compute the expectation of the loss (1), as in [34]. The sampling process does not interfere with computing the gradient with respect to $\theta$. However, the gradient computation with respect to $\phi$ is not feasible when the parameters are integrated with the random samples.

To eliminate this problem, [9] introduced the following reparametrization trick. Assuming $q_{\phi }(z|x)$ is a form of a Gaussian variable, the variable *z* can be written as $z = \varvec{\mu }_{\phi }(x) + \varvec{\sigma }_{\phi }(x) \odot \varvec{\epsilon }$ where $\varvec{\epsilon } \sim N(0,1)$ and $\odot$ denote the element-wise product. Now, we obtain

2 $$\begin{aligned} \nabla _{\phi }\mathbb{E}_{q_{\phi }(z|x)}\log p_{\theta }(x|z)&= \nabla _{\phi }\mathbb{E}_{\varvec{\epsilon } \sim N(0,1)}\log p_{\theta }(x|\varvec{\mu }_{\phi }(x) + \varvec{\sigma }_{\phi }(x) \odot \varvec{\epsilon }) \nonumber \\&= \mathbb{E}_{\varvec{\epsilon } \sim N(0,1)} \nabla _{\phi } \log p_{\theta }(x|\varvec{\mu }_{\phi }(x) + \varvec{\sigma }_{\phi }(x) \odot \varvec{\epsilon }) \end{aligned}$$

The last expectation is still subject to the law of the auxiliary variable $\varvec{\epsilon }$, so we can apply the Monte Carlo approximation to the Eq. (2). Therefore, in practice, the VAE is trained with the reparametrization trick. For more details, see [9]. Here, $\varvec{\mu }_{\phi }$ and $\varvec{\sigma }_{\phi }$ take the form of MLPs, called encoder networks. The likelihood $p_{\theta }(x|z)$ plays the role of the decoder. The VAE algorithm is shown in Algorithm 1.

**Figure:**
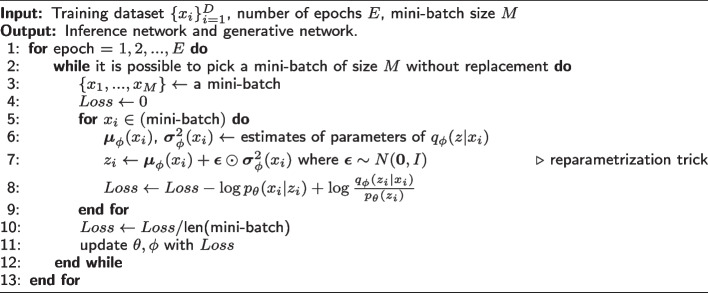
**Algorithm 1** Algorithm of VAE

### UMAP

UMAP is one of the most popular dimension reduction algorithms in the data-science field [35–38]. The algorithm consists of two primary steps: computing the similarity weights among the data and finding lower-dimensional embeddings that best match the computed similarity weights. What follows is a specific description of the UMAP. We denote the *k*-nearest neighbors by $\lbrace x_{i_{j}} \rbrace _{j=1}^{k}$ for each observation $x_{i}$. The distance between observations is transformed to the following weight

$$\begin{aligned} w(x_{i},x_{j}) = {\left\{ \begin{array}{ll} \exp \left( \frac{-\max (0, d(x_{i},x_{j}) - \rho _{i})}{\sigma _{i}}\right) &{} \text{for } j \in \lbrace i_{1},...,i_{k} \rbrace \\ 0 &{} \text{otherwise}, \end{array}\right. } \end{aligned}$$

where $\rho _{i}$ is the positive minimum distance from $x_{i}$, and $\sigma _{i}$ is the normalizing factor. The symmetrized weight $\mu _{ij} = w(x_{i},x_{j}) + w(x_{j},x_{i}) - w(x_{i},x_{j})w(x_{j},x_{i})$ becomes the target similarity between $x_{i}$ and $x_{j}$. In the optimization step, the following objective function is solved

3 $$\begin{aligned} \min _{\{y_i\}_{i=1}^n} -\sum _{i} \sum _{j \ne i} \left( \mu _{ij}\ln \left( \frac{1}{1+a\Vert y_{i} - y_{j} \Vert ^{2b}}\right) + (1-\mu _{ij})\ln \left( 1-\frac{1}{1+a\Vert y_{i} - y_{j} \Vert ^{2b}}\right) \right) , \end{aligned}$$

where *a* and *b* are hyperparameters. The UMAP finds the embedding to best reproduce the target similarities $\mu _{ij}$.

The optimization is not scalable for large data, because the computational complexity of (3) is proportional to the square of the size of the data due to the double summation. The following sampling scheme [39] is used to relieve the computational problem. For an embedding $y_{i}$, one neighbor $y_j$ with $\mu _{ij}>0$ is sampled. The sample is called a positive sample. Additionally, $M \ll N$ embeddings are randomly sampled; these are called negative samples, and are used when the double summation of (3) is computed. The use of negative samples reduces the computational cost of the algorithm to *O*(*NM*). Further details for the optimization step can be found in [14, 40].

### saVAE

**Fig. 10:**
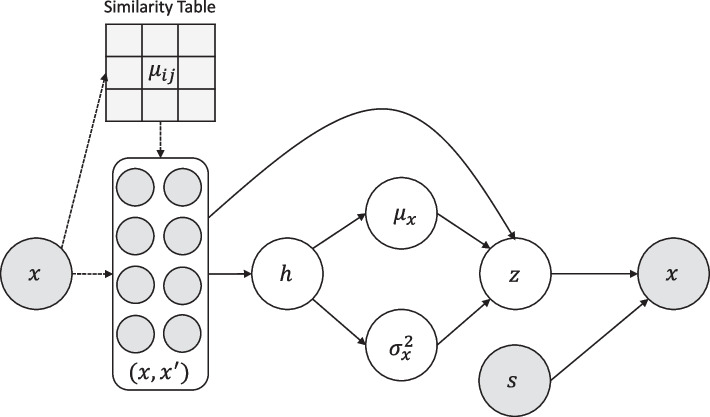
Graphical model of our saVAE. All plain arrows are multi-layered perceptrons, except for the curved one. The curved arrow reflects the similarities among positive samples in the latent space. The dotted arrows represent the process of making the paired samples according to the precalculated similarity table of the observed data. The gray circles are observable; the white ones are not. The variable *z* is computed using the reparametrization trick [9], that is, $z = \varvec{\mu } + \varvec{\epsilon } \odot \varvec{\sigma }$, where $\varvec{\epsilon } \sim N(\varvec{0},I)$. Here, *h* is a hidden variable, and *s* is an optional input. See Methods section

Our saVAE approach bridges VAE and UMAP using a regularization framework. Directly combining the VAE objective with the UMAP loss is not feasible for the following three reasons. First, the optimization arguments of VAE and UMAP are not the same because VAE optimizes its MLP parameters, while UMAP finds lower dimensional embeddings. Second, in the stochastic gradient algorithm, VAE uses a single data point, whereas UMAP uses a pair of data points. Third, the algorithmic convergence rates of the two are quite different, requiring careful balancing.

To address the first problem, we make the UMAP loss a function of the MLP parameters shared with the VAE. Specifically, assuming the embeddings are from the variational distribution, the lower dimensional similarities are replaced with their expectations $q_{\phi }(z|x)$. Our loss function is then as follows:

4 $$\begin{aligned}{} & {} -\mathbb{E}_{q_{\phi }(z|x)} \log p_{\theta }(x|z) + \mathbb{E}_{q_{\phi }(z|x)} \log \frac{q_{\phi }(z|x)}{p_{\theta }(z)} \nonumber \\{} & {} \quad -\lambda \sum _{i}\sum _{j \ne i} \mu _{ij}\ln (\tilde{\nu }_{ij}) + (1-\mu _{ij})\ln (1-\tilde{\nu }_{ij}), \end{aligned}$$

where $\tilde{\nu }_{ij} = \left[ \mathbb{E}_{q_{\phi }(z|x_{i})}\mathbb{E}_{q_{\phi }(z'|x_{j})}\frac{1}{1+a\Vert z - z' \Vert ^{2b}} \right]$ and $\lambda (>0)$ is a weight.

Here, the influence on the parameters of the conditional density function of *z*|*x* is informed by the precomputed high dimensional similarities $\mu _{ij}$. The similarity information then affects the VAE inference when the parameters are shared. The regularizer not only makes a more well-grouped representation of the data but also assists the generator $p_{\theta }(x|z)$ to reconstruct better with reliable similarity information. If the similarity is not reliable, the regularization hurts the reconstruction performance, and the inference network will correct the variational distribution to modify the embeddings. In either case, the variational distribution is adjusted to make the loss (4) decrease. Hence, the two loss functions will have positive influences on each other during the training. Our model is visualized in Fig. 10.

The second problem is resolved by the following implementation. In the application of the stochastic gradient descent algorithm, the VAE is usually trained using a mini-batch, whereas the UMAP is based on sampling (especially pairs). So there is no natural way to implement them simultaneously. To resolve this problem, for each data point $x_{i}$ in a mini-batch, we sample $P_{i}$ positive data $(x_{i}^{1},...,x_{i}^{P_{i}})$, where $P_i$ is the number of positive samples, according to the precalculated similarities with $x_{i}$; we then concatenate $x_{i}$ with $x_{i}^{j}$ so that we obtain a paired dataset $\lbrace (x_{i},x_{i}^{j})\rbrace _{j=1}^{P_{i}}$, which can be used as input for the UMAP. In this case, the VAE inference is made using the unpacked data by treating $\lbrace x_{i},x_{i}^{1},x_{i},x_{i}^{2},...,x_{i},x_{i}^{P_{i}}\rbrace$ as a mini-batch. In contrast to the UMAP, the negative samples of $x_{i}$ are randomly taken from the augmented mini-batch $\lbrace x_{i}^{j}\rbrace _{i,j}$.

The third problem is solved by the selection of an appropriate $\lambda$ in (4) and the introduction of parameter update frequencies. Specifically, we select $\lambda$ as the largest value that decreases the VAE loss. Motivated by [41], we further introduce the pre-defined number *I*, which has the role of controlling the update frequencies. That is, the UMAP loss is updated *I* times per update of the VAE loss. This value strikes a balance between VAE and UMAP, and reduces the training time. With these two hyperparameters $\lambda$ and *I*, the convergence rates of the two losses become similar. Our saVAE algorithm is presented in Algorithm 2.

The specific probability density functions adopted in our method are as follows.

5 $$\begin{aligned} p_{\theta }(z)&= N(\varvec{0},I), \end{aligned}$$

6 $$\begin{aligned} p_{\theta }(x|z)&= {\left\{ \begin{array}{ll} \text{Bernoulli}(\varvec{\mu }_{\theta }(z)) &{} \text{or} \\ N(\varvec{\mu }_{\theta }(z),I) &{} \text{or} \\ \text{NegativeBinomial}(\varvec{\mu }_{\theta }(z), \varvec{r}) \end{array}\right. } \end{aligned}$$

7 $$\begin{aligned} q_{\phi }(z|x)&= N(z; \varvec{\mu }_{\phi }(x),\text{diag}[\varvec{\sigma }^{2}_{\phi }(x)]) \end{aligned}$$

where the bold symbols denote the vector or pre-defined parameters and the sub-indexed symbols are the neural network parameters. The distribution of $p_{\theta }(x|z)$ is dependent on the datatypes, which include Bernoulli, Gaussian, and Negative Binomial distributions. When the prior and variational distributions are assumed to be the Gaussian distribution of (5) and (7), the negative KL divergence can be computed as follows:

$$\begin{aligned} -D_{\text{KL}}(p_{\theta }(z) \parallel q_{\phi }(z|x)) = \frac{1}{2}\sum _{j=1}^{J} \big [ 1 + \log (\varvec{\sigma }^{2}_{\phi }(x))_{j} - (\varvec{\mu }^{2}_{\phi }(x))_{j} - (\varvec{\sigma }^{2}_{\phi }(x))_{j} \big ] \end{aligned}$$

where *J* is the dimension of the latent space and $(\cdot )_{j}$ is the *j*th component.

**Figure:**
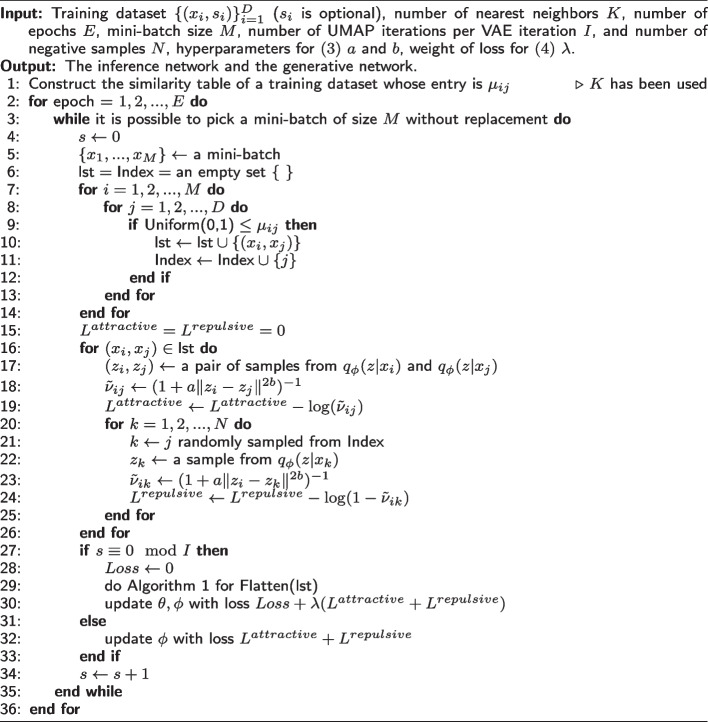
**Algorithm 2** Algorithm of saVAE

### saCVAE

In biological applications including single-cell RNA sequencing data analyses, to find a meaningful latent representation, it is important to adjust for additional covariates. For example, hundreds of cells are collected from a donor, and accounting for the donor-to-donor variations is crucial in the dimension reduction step. Other sources of variability include laboratory conditions, individual characteristics, and instruments used in experiments. These batch-specific effects need to be adjusted to yield an informative latent representation.

**Fig. 11:**
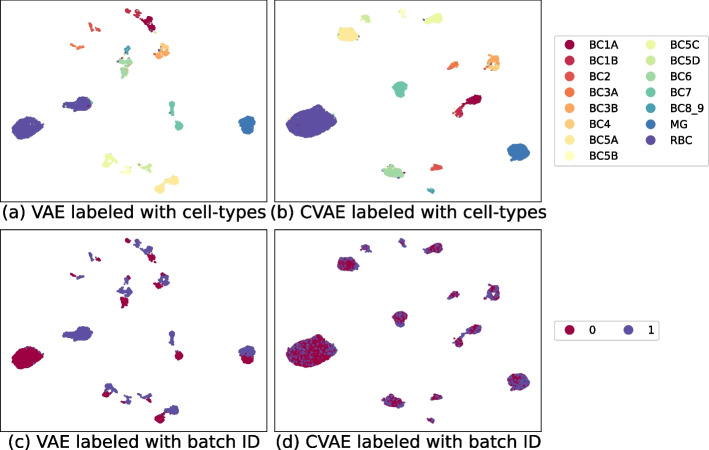
Embeddings from retina dataset. VAE and CVAE are applied to the retina dataset. From **a** and **c**, we find that the retina dataset is subject to a covariate effect, which causes additional groupings beyond the cell types. As shown in **b** and **d**, after the correction with CVAE, the covariate effect is well resolved. In **c** and **d**, the labels 0 and 1 represent the batch ID

The CVAE [42] accounts for such covariate effects. Specifically, for the provided covariate *s*, the loss function of the CVAE is given by

8 $$\begin{aligned} -\mathbb{E}_{q_{\phi }(z|x,s)} \log p_{\theta }(x|z,s) + \mathbb{E}_{q_{\phi }(z|x,s)} \log \frac{q_{\phi }(z|x,s)}{p_{\theta }(z)}. \end{aligned}$$

The notation |*s* means that the given covariate *s*, the inputs of the neural network is concatenated into *s*. If it is categorical, we transformed it into a one-hot encoded vector before inputting the network. After training, a fixed value $s_0$ is used for a covariate shift; in other words, a sample from the distribution $q_{\phi }(z|x, s_0)$ is used as a corrected sample. For example, in Fig. 11, we present the latent representations without batch correction using single-cell RNA sequencing data obtained from the retina. We then use CVAE to estimate the conditional distribution for the covariate shift and visualize the latent representations after adjusting for the covariate effects. The additional separation disappears, suggesting that the grouping comes from the covariate differences. From our experiments across diverse datasets, we found that the CVAE successfully eliminates variabilities from the covariates. We also found that the batch correction is more effective when we use $q_{\phi }(z|x)$ in (8) instead of $q_{\phi }(z|x, s_0)$. It appears that covariate shift constantly occurs during the training of $q_{\phi }(z|x)$. In our paper, we used $q_{\phi }(z|x)$ to adjust for covariate effects.

Replacing the VAE loss function with CVAE loss alone does not solve the covariate problem in our framework, because the similarity weights in the UMAP loss function are still subject to covariate effects. To solve this problem, we propose a two-stage approach in which similarity weights are computed via the covariate-adjusted embeddings from the first round CVAE and, in the second step, the CVAE with the UMAP loss function is trained. Our final objective function of the saCVAE is given as follows:

$$\begin{aligned}{} & {} -\mathbb{E}_{q_{\phi }(z|x)} \log p_{\theta }(x|z,s) + \mathbb{E}_{q_{\phi }(z|x)} \log \frac{q_{\phi }(z|x)}{p_{\theta }(z)} \nonumber \\{} & {} -\lambda \sum _{i}\sum _{j \ne i} \tilde{\mu }_{ij}\ln (\tilde{\nu }_{ij}) + (1- \tilde{\mu }_{ij})\ln (1-\tilde{\nu }_{ij}), \end{aligned}$$

where $\tilde{\mu }_{ij}$ is the weight computed from the covariate-adjusted embeddings and $\tilde{\nu }_{ij}$ and $\lambda$ are defined in the previous section.

## Related works

To the best of our knowledge, the first attempt at bridging the VAE to similarity-based dimension reduction was variational manifold probabilistic linear discriminant analysis (vm-PLDA) [43]. However, the probability model for the data was limited to linear factor models that cannot be easily extended to other discrete datasets. Later, scvis [18] used the objective of non-linear dimensional reduction to regularize the latent space of VAEs. More precisely, it captured underlying low-dimensional structures in a given dataset by imposing the t-SNE objective on the latent space of the VAEs to achieve a more compressed representation of the data. Because this method employs parametric mapping from the high-dimensional space to a low-dimensional embedding, it is possible to naturally add new data points to a learned latent space. On the other hand, this method still inherits the limitations of t-SNE. In addition, [16] proposed deep clustering via a Gaussian-mixture variational autoencoder with graph embedding (DGG); this method inferred the latent representation using the VaDE model with distance regularization. The authors selected the Jenson-Shannon (JS) divergence as a distance between two density functions. The computation, however, was not feasible, so they relaxed it to its upper bound to promote computational efficiency. Additionally, they trained a Siamese network [44, 45] to better learn the similarities among the observed data. This led to robust and excellent performance. One problem in DGG is that it is not equipped with a controlling parameter because the upper bound is derived under only one particular set-up.

In scRNA-seq analysis, VAE-based models have successfully performed many tasks, such as imputation, batch correction, clustering, dimension reduction, and prediction [4–6, 8, 18, 46–48]. Dhaka [46] is one of the standard VAE-based methods for scRNA data, specializing in tumor cell identification. In scVAE [5], the variational approximation is improved by using a mixture of Gaussian distributions. Yet the number of mixture components needs to be optimally selected, which may be challenging in actual application. VASC [4] introduced a zero-inflated layer into VAE for modeling dropout events in scRNA data. Empirically, however, the estimates exhibited large variabilities. In netAE [47], a VAE-based cell-type prediction model was developed, which utilizes the modularity measures. The approach requires a well trained classifier to guarantee its efficiency. A specialized method, bmVAE [48], is proposed for genotype prediction by integrating VAE, GMM, and Gibbs sampling. Its actual application requires special attention because doublets or triplets may appear in tumor data. Finally, scVI [6] is a versatile tool for various tasks and commonly used as a baseline model in [49]. As a result, performance in each task requires data-specific adjustments.

### Transcriptome data preprocessing

Because these transcriptomes have many features (or genes), the outlier removal and feature selection were conducted via the HVGs method [26]. We used 1200 screened features (see Additional file 1: section B for more discussions). We use the Negative Binomial distribution (6) because it is a common assumption of single-cell RNA-sequencing modeling. As a variational distribution, we used $q_{\phi }(z|x) = N(z; \varvec{\mu }_{\phi }(\log (1+x)),\text{diag}[\varvec{\sigma }^{2}_{\phi }(\log (1+x))])$). We found that this particular choice produced the best results throughout the experiments. For the retina and heart cell atlas, some covariate information is available. We used the CVAE to adjust for covariates, as described in the saCVAE section.

### Implementation details

**Table 1:** Hypermarameters used in the model

Hyperparameters	Datasets					
	Synthetic	MNIST	Cortex	PBMC	Retina	Heart cell atlas
Distribution	Gaussian	Binoimal	NB	NB	NB	NB
Latent dimension	2	10	10	10	10	10
Hidden dimension	20	512	512	512	512	512
Weight $\lambda$	$10^{3}$	$10^{3}$	$10^{3}$	$10^{3}$	$10^{3}$	$10^{3}$
Iteration ratio *I*	5	5	5	5	5	5
Covariate effects	No	No	No	No	Yes	Yes
(CVAE) Hidden dimension	None	None	None	None	128	128

The choice of $\lambda$ and *I* is discussed on Additional file 1: section C

In the implementation, the multi-layered perceptrons used in the VAE consist of three fully connected layers with ReLU [50] activation. The multi-layered perceptrons in the CVAE have only one fully-connected layer with ReLU, BatchNormalization [51], and Dropout [52]. The expectation in Equation (4) was computed via the Monte Carlo method with a single sample [33], and the network weights were trained using the Adam optimizer [53] with the learning rate of 0.001 and the batch size of 128. The parameters in UMAP are the number of nearest neighbors and the min-dist, which are specified in the lower-dimensional similarity functions. We set $K=30$ and $\text{ min-dist } = 0$, which showed the best performance in our experiments. Table 1 summarizes the hyperparameters used in our experiments.

For real-world datasets, our model was compared to UMAP^1^ [14], DGG^2^ [16], scvis^3^ [18], Ivis^4^ [20], and scVI^5^[6, 19]. These baseline models were utilized to find latent representations of the various datasets.

The MNIST dataset is different from other single-cell RNA sequencing datasets in its likelihood specification. The available DGG code works only on the MNIST dataset (because DGG exploits a pre-trained VaDE and the author provided only the trained parameters of the MNIST without the training code), while the scVI code cannot be used for the MNIST dataset. When not available, those methods are not included in the comparison. Because the retina and heart cell atlas datasets are subject to covariate effects, we compared our methods with scVI because it is the only other method that is equipped with covariate adjustment. Lastly, the implementation of the scvis was too outdated to be modified: to alleviate the computational burden, we used PCA to reduce the dimension of the features to 100. We also fixed the degree of freedom of the t-distribution at one; the t-distribution was used as the similarity criterion of the scvis method.

For a fair comparison, all methods were trained to learn ten-dimensional latent embeddings. We set the hyperparameters of the UMAP to be the same as those of saVAE by setting $K = 30$ and $\text{ min-dist } = 0$. scVI supports many implementation options. We set the likelihood and library size availability at NB and False, respectively. The rest of the baseline methods’ hyperparameters were set according to the suggested default numbers.

To visualize the latent embeddings in the 2d plots, we ran UMAP again with the default parameters ($K = 15$ and $\text{ min-dist } = 0.1$). However, we found that the spectral embedding [54], suggested as a default initialization of UMAP, was not suitable for this type of dimension reduction. On the other hand, PCA [55] shows fast convergence of UMAP. We thus used PCA initialization for our implementation.

### Supplementary Information

**Additional file 1**. The PBMC dataset has only negligible batch effects. When varying the HVG selection criteria to 900, 1200, 1500, and 3000, the performance of our method was not significantly affected on any dataset. Finally, a sensitivity analysis of the hyperparameters shows that although other hyperparameter values provide better results, the values we chose also yield strong performance.

## Data Availability

The datasets and source code are available at https://github.com/GwangWooKim/saVAE.

## Abbreviations

VAE: Variational autoencoder
t-SNE: T-distributed stochastic neighbor embedding
UMAP: Uniform manifold approximation and projection
CVAE: Conditional VAE
GAN: Generative adversarial network
saVAE: Similarity-assisted variational autoencoder
saCVAE: Similarity-assisted conditional variational autoencoder
MLP: Multi-layered perceptron
PBMC: Peripheral blood mononuclear cell
ARI: Adjusted Rand Index
NMI: Normalized mutual information

## Footnotes

1 The implementation is available at https://github.com/lmcinnes/umap.

2 https://github.com/ngoc-nguyen-0/DGG.

3 https://github.com/shahcompbio/scvis.

4 https://github.com/beringresearch/ivis.

5 https://github.com/scverse/scvi-tools.

## References

[1] Salakhutdinov R (2015). Learning deep generative models. Annu Rev Stat Appl.

[2] Bond-Taylor S, Leach A, Long Y, Willcocks CG (2022). Deep generative modelling: a comparative review of VAEs, GANs, normalizing flows, energy-based and autoregressive models. IEEE Trans Pattern Anal Mach Intell.

[3] Gm H, Gourisaria MK, Pandey M, Rautaray SS (2020). A comprehensive survey and analysis of generative models in machine learning. Comput Sci Rev.

[4] Wang D, Gu J (2018). VASC: dimension reduction and visualization of single-cell RNA-seq data by deep variational autoencoder. Genom Proteom Bioinform.

[5] Grønbech CH, Vording MF, Timshel PN, Sønderby CK, Pers TH, Winther O (2020). scVAE: variational auto-encoders for single-cell gene expression data. Bioinformatics.

[6] Lopez R, Regier J, Cole MB, Jordan MI, Yosef N (2018). Deep generative modeling for single-cell transcriptomics. Nat Methods.

[7] Way GP, Greene CS. Extracting a biologically relevant latent space from cancer transcriptomes with variational autoencoders. In: Biocomputing. World Scientific. 2018; pp. 80–91. PMC5728678 29218871

[8] Simidjievski N, Bodnar C, Tariq I, Scherer P, Andres Terre H, Shams Z, Jamnik M, Liò P (2019). Variational autoencoders for cancer data integration: design principles and computational practice. Front Genet.

[9] Kingma DP, Welling M. Auto-encoding variational Bayes. In: Bengio Y, LeCun Y, editors. 2nd international conference on learning representations, ICLR 2014, Banff, AB, Canada, April 14–16, 2014, Conference Track Proceedings. 2014.

[10] Goodfellow I, Pouget-Abadie J, Mirza M, Xu B, Warde-Farley D, Ozair S, Courville A, Bengio Y. Generative adversarial nets. In: Ghahramani Z, Welling M, Cortes C, Lawrence N, Weinberger KQ, editors. Advances in neural information processing systems. 2014; vol. 27.

[11] Donahue J, Krähenbühl P, Darrell T. Adversarial feature learning. In: 5th international conference on learning representations, ICLR 2017, Toulon, France, April 24–26, 2017, Conference Track Proceedings. 2017.

[12] Mukherjee S, Asnani H, Lin E, Kannan S (2019). ClusterGAN: latent space clustering in generative adversarial networks. Proc AAAI Conf Artif Intell.

[13] van der Maaten L, Hinton G (2008). Visualizing data using t-SNE. J Mach Learn Res.

[14] McInnes L, Healy J, Saul N, Grossberger L (2018). UMAP: uniform manifold approximation and projection. J Open Source Softw.

[15] Buitinck L, Louppe G, Blondel M, Pedregosa F, Mueller A, Grisel O, Niculae V, Prettenhofer P, Gramfort A, Grobler J, Layton R, VanderPlas J, Joly A, Holt B, Varoquaux G. API design for machine learning software: experiences from the scikit-learn project. In: ECML PKDD workshop: languages for data mining and machine learning. 2013; pp. 108–122.

[16] Yang L, Cheung NM, Li J, Fang J. Deep Clustering by Gaussian Mixture Variational Autoencoders With Graph Embedding. In: Proceedings of the IEEE/CVF International Conference on Computer Vision (ICCV). 2019.

[17] Jiang Z, Zheng Y, Tan H, Tang B, Zhou H. Variational deep embedding: an unsupervised and generative approach to clustering. In: Proceedings of the 26th international joint conference on artificial intelligence. 2017; pp. 1965–1972.

[18] Ding J, Condon A, Shah SP (2018). Interpretable dimensionality reduction of single cell transcriptome data with deep generative models. Nat Commun.

[19] Gayoso A, Lopez R, Xing G, Boyeau P, Valiollah Pour Amiri V, Hong J, Wu K, Jayasuriya M, Mehlman E, Langevin M, Liu Y, Samaran J, Misrachi G, Nazaret A, Clivio O, Xu C, Ashuach T, Gabitto M, Lotfollahi M, Svensson V, da Veiga Beltrame E, Kleshchevnikov V, Talavera-López C, Pachter L, Theis FJ, Streets A, Jordan MI, Regier J, Yosef N (2022). A python library for probabilistic analysis of single-cell omics data. Nat Biotechnol..

[20] Szubert B, Cole JE, Monaco C, Drozdov I (2019). Structure-preserving visualisation of high dimensional single-cell datasets. Sci Rep.

[21] Zeisel A, Muñoz-Manchado AB, Codeluppi S, Lönnerberg P, Manno GL, Juréus A, Marques S, Munguba H, He L, Betsholtz C, Rolny C, Castelo-Branco G, Hjerling-Leffler J, Linnarsson S (2015). Cell types in the mouse cortex and hippocampus revealed by single-cell RNA-seq. Science.

[22] Zheng GX, Terry JM, Belgrader P, Ryvkin P, Bent ZW, Wilson R, Ziraldo SB, Wheeler TD, McDermott GP, Zhu J (2017). Massively parallel digital transcriptional profiling of single cells. Nat Commun.

[23] Tuller T, Atar S, Ruppin E, Gurevich M, Achiron A (2013). Common and specific signatures of gene expression and protein–protein interactions in autoimmune diseases. Genes Immunity.

[24] Beer L, Mildner M, Gyöngyösi M, Ankersmit HJ (2016). Peripheral blood mononuclear cell secretome for tissue repair. Apoptosis.

[25] Eriksson A, Österroos A, Hassan S, Gullbo J, Rickardson L, Jarvius M, Nygren P, Fryknäs M, Höglund M, Larsson R (2015). Drug screen in patient cells suggests quinacrine to be repositioned for treatment of acute myeloid leukemia. Blood Cancer J.

[26] Stuart T, Butler A, Hoffman P, Hafemeister C, Papalexi E, Mauck WM, Hao Y, Stoeckius M, Smibert P, Satija R (2019). Comprehensive integration of single-cell data. Cell.

[27] Lloyd S (1982). Least squares quantization in PCM. IEEE Trans Inf Theory.

[28] Ester M, Kriegel HP, Sander J, Xu X. A density-based algorithm for discovering clusters in large spatial databases with noise. In: Proceedings of the second international conference on knowledge discovery and data mining. 1996; pp. 226–231

[29] Hubert L, Arabie P (1985). Comparing partitions. J Classif.

[30] Vinh NX, Epps J, Bailey J. Information theoretic measures for clusterings comparison: is a correction for chance necessary? In: Proceedings of the 26th annual international conference on machine learning. 2009; pp. 1073–1080.

[31] Shekhar K, Lapan SW, Whitney IE, Tran NM, Macosko EZ, Kowalczyk M, Adiconis X, Levin JZ, Nemesh J, Goldman M, McCarroll SA, Cepko CL, Regev A, Sanes JR (2016). Comprehensive classification of retinal bipolar neurons by single-cell transcriptomics. Cell.

[32] Litviňuková M, Talavera-López C, Maatz H, Reichart D, Worth CL, Lindberg EL, Kanda M, Polanski K, Heinig M, Lee M (2020). Cells of the adult human heart. Nature.

[33] Metropolis N, Ulam S (1949). The Monte Carlo method. J Am Stat Assoc.

[34] Spall JC (2005). Introduction to stochastic search and optimization: estimation, simulation, and control.

[35] Dorrity MW, Saunders LM, Queitsch C, Fields S, Trapnell C (2020). Dimensionality reduction by umap to visualize physical and genetic interactions. Nat Commun.

[36] Allaoui M, Kherfi ML, Cheriet A. Considerably improving clustering algorithms using UMAP dimensionality reduction technique: a comparative study. In: El Moataz A, Mammass D, Mansouri A, Nouboud F, editors. Image and signal processing. Springer. 2020; pp. 317–325.

[37] Vermeulen M, Smith K, Eremin K, Rayner G, Walton M (2021). Application of uniform manifold approximation and projection (UMAP) in spectral imaging of artworks. Spectrochim Acta Part A Mol Biomol Spectrosc.

[38] Milošević D, Medeiros AS, Stojković Piperac M, Cvijanović D, Soininen J, Milosavljević A, Predić B (2022). The application of uniform manifold approximation and projection (UMAP) for unconstrained ordination and classification of biological indicators in aquatic ecology. Sci Total Environ.

[39] Mikolov T, Sutskever I, Chen K, Corrado GS, Dean J. Distributed representations of words and phrases and their compositionality. In: Burges CJ, Bottou L, Welling M, Ghahramani Z, Weinberger KQ, editors. Advances in neural information processing systems, 2013; vol. 26.

[40] Ghojogh B, Ghodsi A, Karray F, Crowley M. Uniform manifold approximation and projection (UMAP) and its variants: tutorial and survey. arXiv preprint. 2021. arXiv:2109.02508

[41] Arjovsky M, Chintala S, Bottou L. Wasserstein generative adversarial networks. In: Precup D, Teh YW, editors. Proceedings of the 34th international conference on machine learning. 2017; vol. 70, pp. 214–223.

[42] Sohn K, Lee H, Yan X. Learning structured output representation using deep conditional generative models. In: Cortes C, Lawrence N, Lee D, Sugiyama M, Garnett R, editors. Advances in neural information processing systems. 2015; vol. 28.

[43] Chien JT, Hsu CW. Variational manifold learning for speaker recognition. In: 2017 IEEE international conference on acoustics, speech and signal processing (ICASSP). 2017; pp. 4935–4939.

[44] Shaham U, Stanton KP, Li H, Basri R, Nadler B, Kluger Y. SpectralNet: spectral clustering using deep neural networks. In: ICLR (Poster). 2018.

[45] Hadsell R, Chopra S, LeCun Y. Dimensionality reduction by learning an invariant mapping. In: 2006 IEEE computer society conference on computer vision and pattern recognition (CVPR’06). 2006; pp. 1735–1742.

[46] Rashid S, Shah S, Bar-Joseph Z, Pandya R (2019). Dhaka: variational autoencoder for unmasking tumor heterogeneity from single cell genomic data. Bioinformatics.

[47] Dong Z, Alterovitz G (2020). netAE: semi-supervised dimensionality reduction of single-cell RNA sequencing to facilitate cell labeling. Bioinformatics.

[48] Yan J, Ma M, Yu Z (2022). bmVAE: a variational autoencoder method for clustering single-cell mutation data. Bioinformatics.

[49] Flores M, Liu Z, Zhang T, Hasib MM, Chiu Y-C, Ye Z, Paniagua K, Jo S, Zhang J, Gao S-J, Jin Y-F, Chen Y, Huang Y (2021). Deep learning tackles single-cell analysis: a survey of deep learning for scRNA-seq analysis. Brief Bioinform.

[50] Agarap AF. Deep learning using rectified linear units (relu). 2018. arXiv preprint arXiv:1803.08375

[51] Ioffe S, Szegedy C. Batch normalization: accelerating deep network training by reducing internal covariate shift. In: Proceedings of the 32nd international conference on machine learning. 2015; pp. 448–456.

[52] Srivastava N, Hinton G, Krizhevsky A, Sutskever I, Salakhutdinov R (2014). Dropout: a simple way to prevent neural networks from overfitting. J Mach Learn Res.

[53] Kingma DP, Ba J. Adam: a method for stochastic optimization. In: Bengio Y, LeCun Y, editors. 3rd International conference on learning representations, ICLR 2015, San Diego, CA, USA, May 7-9, 2015, Conference Track Proceedings. 2015. arXiv: 1412.6980

[54] Belkin M, Niyogi P (2003). Laplacian eigenmaps for dimensionality reduction and data representation. Neural Comput.

[55] FRS, KP. LIII. On lines and planes of closest fit to systems of points in space. The London, Edinburgh, and Dublin Philosophical Magazine and Journal of Science 2. 1901; 559–572.

