# Supplementary material for: Similarity-assisted variational autoencoder for nonlinear dimension reduction with application to single-cell RNA sequencing data
Source: BMC Bioinformatics. 2023 Nov 14;24:432. doi: 10.1186/s12859-023-05552-1 (PMC10647110; doi:10.1186/s12859-023-05552-1)
Supplement: Supplementary file 1 — Additional file 1. The PBMC dataset has only negligible batch effects. When varying the HVG selection criteria to 900, 1200, 1500, and 3000, the performance of our method was not significantly affected on any dataset. Finally, a sensitivity analysis of the hyperparameters shows that although other hyperparameter values provide better results, the values we chose also yield strong performance. [file 12859_2023_5552_MOESM1_ESM.pdf]

Supplementary Materials for Similarity-assisted Variational Autoencoder for Nonlinear Dimension Reduction with Application to Single-cell RNA Sequencing Data

Gwangwoo Kim[*], Hyonho Chun[†]

A Batch effect of the PBMC data

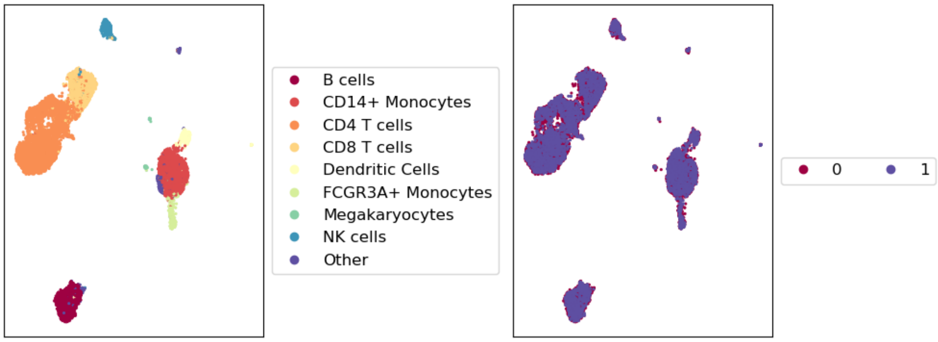

(a) VAE labeled with cell-types (b) VAE labeled with batch ID

Figure S1: Embeddings from PBMC data. In (b), 0 and 1 represent the batch IDs.

To demonstrate that PBMC data does not have a batch effect, we trained the VAE without batch information. We used the trained inference network to obtain embeddings. Figure S1 shows that there is no batch effect in this dataset.

[*]Graduate School of Data Science, Korea Advanced Institute of Science and Technology (KAIST), urikokp@kaist.ac.kr

[†]Department of Mathematical Sciences, Korea Advanced Institute of Science and Technology (KAIST), hyonhochun@kaist.ac.kr

B Discussion on the number of screened genes

Due to the dimensionality of scRNA sequencing data, we screened genes via the Highly Variable Genes (HVG) [1] selection method. The HVG screening uses only the top n highest varying genes. The authors of [2] used the value of 1,200 for n and we followed this suggestion for fair comparison.

We first present the ordered gene variances in Figure S2. The cut-off value of 1,200 covers the majority of highly varying genes across four datasets used in our study. We then study the influence of the screening cut-off value on the performance of our approach. We varied it to 900, 1,200, 1,500, 2,000, and 3,000 genes and measured the performance using ARI and NMI (Table S1). Except for the PBMC dataset, more features make it perform better, but the improvement tends to be mild. In the PBMC dataset our method performs worse with more features, implying the lowly varying genes may be noise variables. We remark that saVAE is utilized for the cortex and the PBMC datasets and saCVAE for the retina and the heart cell atlas datasets. In sum, the cut-off value for the feature screening has an influence on the performance of our approach, where the optimal cut-off value is data-specific. We do not pursue the feature selection further as our method is not for the feature selection. We used the value of $n = 1,200$ following [1] for fair comparison.

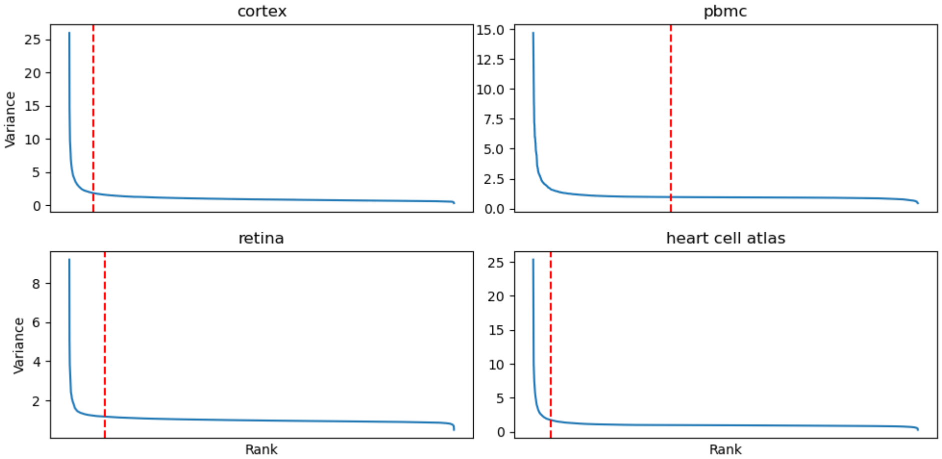

Figure S2: Gene variances are presented in decreasing order. Note that the blue curve is a continuation of a discrete bar plot (because there were too many genes). The red dashed vertical line is the position of the 1200th highly varying gene.

		n=900	n=1200	n=1500	n=2000	n=3000
cortex	ARI	0.77	0.79	0.80	0.81	0.87
	NMI	0.75	0.77	0.78	0.80	0.85
PBMC	ARI	0.84	0.84	0.68	0.66	0.66
	NMI	0.81	0.80	0.74	0.74	0.74
retina	ARI	0.97	0.97	0.98	0.98	0.98
	NMI	0.94	0.95	0.95	0.95	0.96
heart cell atlas	ARI	0.91	0.93	0.93	0.92	0.93
	NMI	0.90	0.92	0.92	0.91	0.92

Table S1: Performance of our approach for n = 900, 1200, 1500, 2000, and 3000.

C Hyperparameter selection of λ and I

In the main text, we tested various λ and I using the MNIST dataset and determined optimal values of $\lambda = 1,000$ and $I = 5$. The selected values were then used across the datasets. In this section, we perform the sensitivity analysis on hyperparameters and present the performance along the various hyperparameter values in Table S2. Our predetermined λ and I values do not achieve the optimum but still give strong results. It also shows that higher values for λ and I lead to better performance. This finding, however, may come from the property of the evaluation metric. The NMI favors better clustering, which is the sole goal of the UMAP objective function. It is our future work to develop a computationally efficient selection criterion for the choice of λ and I.

		$\lambda = 10^0$	$\lambda = 10^1$	$\lambda = 10^2$	$\lambda = 10^3$	$\lambda = 10^4$	$\lambda = 10^5$
cortex	$I = 1$	0.24	0.20	0.51	0.77	0.77	0.76
	$I = 5$	0.45	0.54	0.72	0.77	0.76	0.77
	$I = 10$	0.48	0.59	0.72	0.77	0.77	0.77
PBMC	$I = 1$	0.43	0.49	0.73	0.77	0.81	0.81
	$I = 5$	0.58	0.73	0.73	0.80	0.80	0.80
	$I = 10$	0.68	0.75	0.78	0.81	0.81	0.80
retina	$I = 1$	0.19	0.39	0.92	0.95	0.95	0.95
	$I = 5$	0.41	0.92	0.95	0.95	0.95	0.95
	$I = 10$	0.75	0.94	0.95	0.95	0.95	0.95
heart cell atlas	$I = 1$	0.08	0.12	0.85	0.90	0.92	0.92
	$I = 5$	0.18	0.79	0.91	0.92	0.92	0.92
	$I = 10$	0.31	0.87	0.92	0.92	0.92	0.92

Table S2: Sensitivity analysis on λ and I.

References

[1] T. Stuart, A. Butler, P. Hoffman, C. Hafemeister, E. Papalexi, W. M. Mauck, Y. Hao, M. Stoeckius, P. Smibert, and R. Satija, "Comprehensive integration of single-cell data," *Cell*, vol. 177, no. 7, pp. 1888–1902, 2019.

[2] A. Gayoso, R. Lopez, G. Xing, P. Boyeau, V. Valiollah Pour Amiri, J. Hong, K. Wu, M. Jayasuriya, E. Mehlman, M. Langevin, *et al.*, "A python library for probabilistic analysis of single-cell omics data," *Nature biotechnology*, vol. 40, no. 2, pp. 163–166, 2022.
